# Subgrade soils characterization data, for correlation of geotechnical variables on urban roads in northern Colombia

**DOI:** 10.1016/j.dib.2020.106095

**Published:** 2020-07-31

**Authors:** Fernando Jove Wilches, Jorge Luis Argoty Burbano, Edilberto Elías Contreras Sierra

**Affiliations:** a Department of Civil Engineering, Universidad de Sucre, Cra. 28 #5 - 267, Puerta Roja, Sincelejo, Sucre, Colombia; b Department of Civil Engineering, Universidad de Nariño, San Juan de Pasto, Nariño, Colombia; c Universidad de Sucre, Sincelejo, Sucre, Colombia

**Keywords:** Soil characterization, Subgrade, CBR, DPC, Standard soil tests

## Abstract

Within the variables relevant to the design of a pavement structure, the subgrade soils should be considered, which must be characterized in order to determine their mechanical properties and their bearing capacity. However, in developing countries such as Colombia, where the resources available for the design phase of a road project are very scarce, simplified and low-cost techniques should be used while delivering fast results, in order to be able to determine the geotechnical characteristics of soils. Therefore, it is necessary to look for correlations between the different geotechnical variables of subgrade soils. This document contains a database with the main physical characteristics of the soils. To collect these data, 46 geotechnical survey were carried out through several urban road sectors located in the city of Sincelejo, northern Colombia. Field tests were carried out with the Dynamic Cone Penetrometer and laboratory tests from undisturbed samples, for the realization of the California Bearing Ratio. Additionally, from disturbed samples, standard soil tests were conduct. The dataset obtained from the characterization of the soils, helps to create correlations between different variables, in such a way that it is possible to obtain bearing capacity parameters, such as CBR and Resilient Modulus, required for pavement design, based on simpler and faster tests such as the Dynamic Cone Penetrometer test, soil particle size analysis, the Atterberg limits or soil moisture content. In addition, these data can be supplemented by future researches in geographical regions with socioeconomic characteristics similar to those of Colombia.

**Specifications Table**

**Table:** 

**Subject**	Civil and Structural Engineering
**Specific subject area**	Soil classification and properties of soils
**Type of data**	Table
**How data were acquired**	Soil samples were collected from geotechnical surveys made in urban road sectors. They were then laboratory-analyzed using standard soil analysis methods. Microsoft Excel 2013.
**Data format**	Raw
	Analyzed
**Parameters for data collection**	Forty-six sites were selected for soil sampling. The chosen sites are part of an urban area with high potential for socioeconomic growth, but which has had little intervention and investment by government authorities and has been little studied in soil characterization issues.
**Description of data collection**	For soil characteristics research, samples were taken at different sites located in the study area and field trials such as DCP were conducted. The samples were then taken to the laboratory and routine analyses were performed to determine some physical and mechanical properties, using standard soil laboratory methods. Soil particle size analysis, moisture content, Atterberg limits and CBR were tested. Finally, the information was processed and organized using the Microsoft Excel spreadsheet program.
**Data source location**	Institution: Universidad de Sucre
	City: Sincelejo
	Region: Sucre
	Country: Colombia
**Data accessibility**	Raw and analyzed data was deposited in the Mendeley repository as Data, v1, 2020. DOI: 10.17632/6×263rcdhd.1
	http://dx.doi.org/10.17632/6×263rcdhd.1
**Related research article**	F. Jove, R. Hernandez, J. Feria, Estimation of a correlation equation between CBR and DCP for silty soils from the MH group in Sincelejo city, Colombia, International Journal of Civil Engineering and Technology (IJCIET), Article ID: IJCIET_10_09_006, pp. 54–59, (2019). http://www.iaeme.com/ijciet/IJCIET_Paper.asp?sno=17967

**Value of the Data**

• This data relates the properties of soils characteristic of a region in northern Colombia. The data are valuable for: (a) to know the soil types characteristic of the area under research, (b) identify and delimit homogeneous geotechnical regions and (c) decrease uncertainty in soil knowledge in this region.
• The data is useful for institutions, researchers and experts involved in projects related to soil characterization for uses in civil, agricultural engineering and territorial and environmental management.
• The data can be used for the implementation of international correlation models, calibrated to the particular conditions of the characteristic soils of the study area, allowing the obtaining of geotechnical parameters of difficult or costly obtaining through routine low-cost tests.
• There are not many databases with this type of information from the region studied, so this dataset provides researchers with an overview of the types of soil in the area.
• The dataset can provide researchers studying the area with valuable information for new investigations, such as supplementing information or making more complex correlation models.

## 1 Data description

**Fig. 1:**
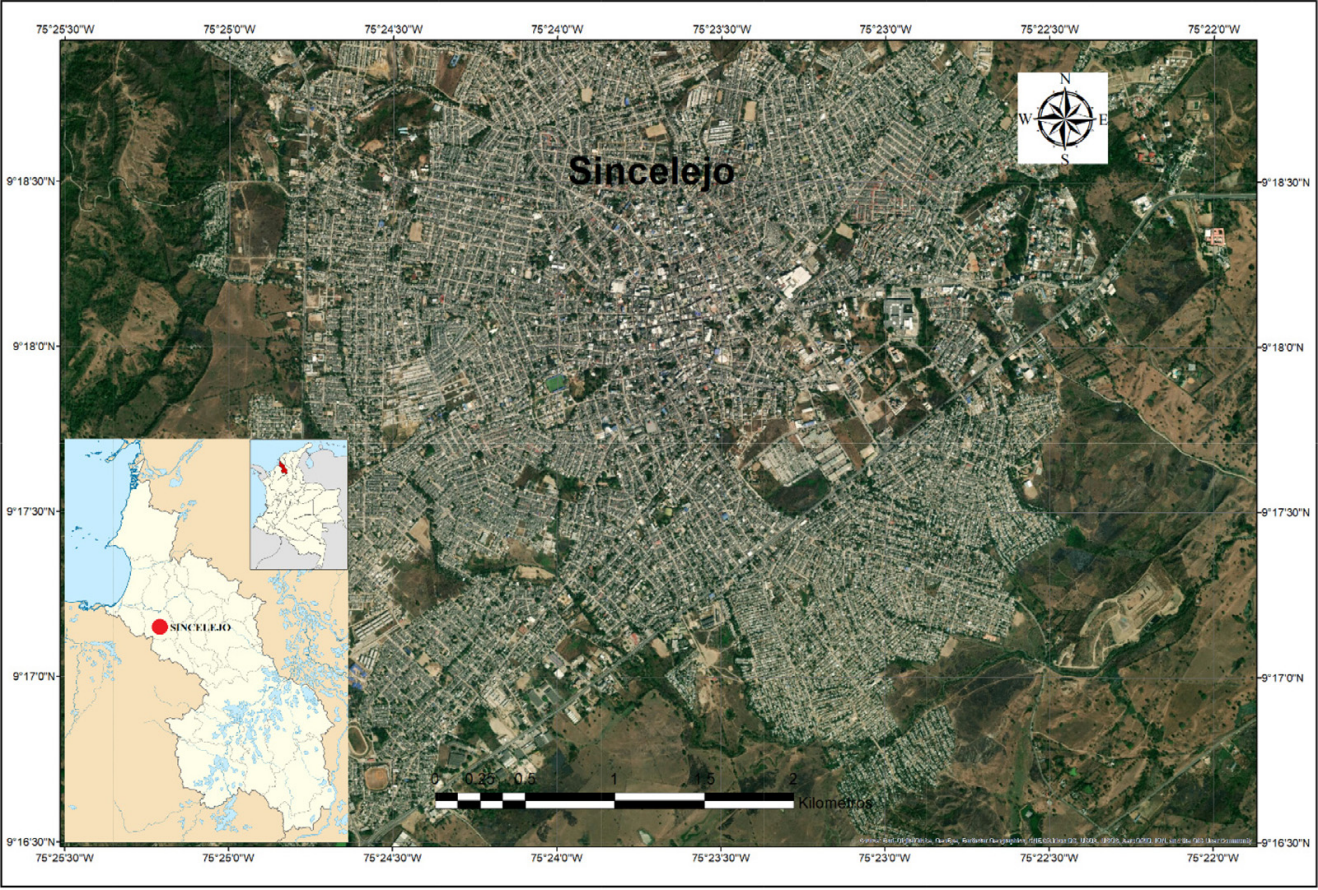
Location of the Sincelejo city (research zone).

**Fig. 2:**
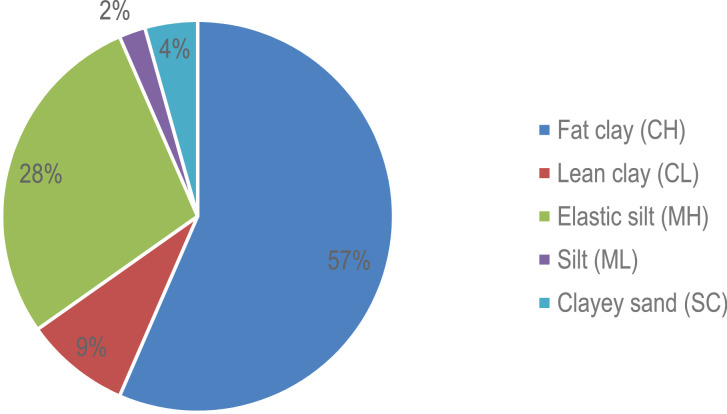
Proportion of subgrade soil types in the study area.

**Fig. 3:**
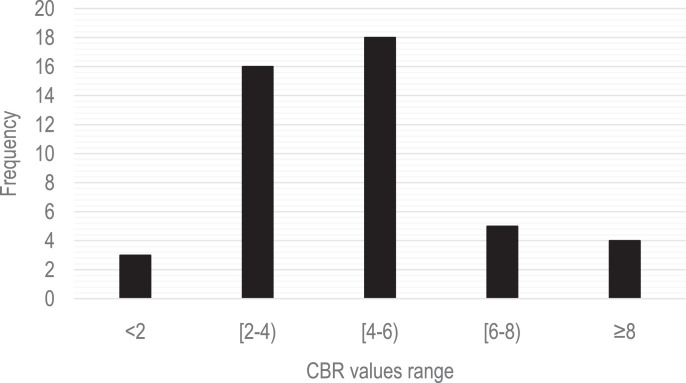
CBR values obtained in the laboratory of the samples tested.

The figures and tables with the physical and mechanical properties of the soils, were obtained and analyzed based on the data obtained from 46 geotechnical surveys located in different sites in the city of Sincelejo, Sucre (Colombia). This section will show the data collected and processed on soil characteristics in the aforementioned region. The raw and processed data files were deposited in the Mendeley data repository DOI: 10.17632/6×263rcdhd.1 http://dx.doi.org/10.17632/6×263rcdhd.1. Fig. 1 shows the location of the research area. This shows the urban area of the city of Sincelejo. Table 1 shows the types of soil found in the area and the number of samples taken in each case. Fig. 2 shows the proportion of soil types found during sampling of the 46 perforations analyzed. In Fig. 3 CBR test results are shown for field samples. Table 2 shows the most representative values for each of the 46 samples, shows the soil classification of each sample (listed in Table 1 and shown in Fig. 2), show the natural moisture content, the soil unit weight as well as the DCPI and CBR values (shown in Fig. 3).

**Table 1:** Characteristic soils found in the area.

Soil classification	Number of samples
Fat clay (CH)	26
Lean clay (CL)	4
Elastic silt (MH)	13
Silt (ML)	1
Clayey sand (SC)	2
Total	46

**Table 2:** Main parameters of soil samples, classification and test results of California Bearing Ratio (CBR) and Dynamic Cone Penetration index (DCP).

Sample	SUCS soil	Soil	Natural moisture			DCPI values	CBR
Sample	classification	Soil	content (%)	Soil unit weights		(mm/blow)	values%
				Ɣh (g/cm³)	Ɣd (g/cm³)		
1	CH	Fat clay	29	1.71	1.33	60.0	2.8
2	MH	Elastic silt	15.1	1.91	1.66	22.5	8.3
3	CH	Fat clay	21.3	1.87	1.54	78.0	3.0
4	MH	Elastic silt	23.5	1.78	1.44	32.4	7.0
5	CH	Fat clay	37.6	1.79	1.30	30.7	5.2
6	MH	Elastic silt	35.5	1.80	1.33	74.0	3.4
7	CH	Fat clay	27.5	1.78	1.40	54.5	2.7
8	MH	Elastic silt	27.5	1.79	1.40	72.0	2.8
9	MH	Elastic silt	27.2	1.61	1.27	20.4	4.8
10	CH	Fat clay	36.7	1.81	1.32	120.0	1.9
11	CH	Fat clay	18.8	1.59	1.34	61.5	3.4
12	CH	Fat clay	19.3	2.09	1.75	43.0	4.2
13	SC	Clayey sand	13.8	2.08	1.83	51.5	4.3
14	CL	Lean clay	13.1	1.99	1.76	34.0	6.0
15	MH	Elastic silt	28.1	1.82	1.42	107.5	2.6
16	CH	Fat clay	26.3	1.81	1.43	55.0	4.0
17	MH	Elastic silt	20.5	1.79	1.49	51.5	5.0
18	CH	Fat clay	26.2	2.09	1.66	37.6	8.7
19	CH	Fat clay	30.8	1.81	1.38	62.5	4.1
20	CL	Lean clay	26.8	1.62	1.28	58.0	3.8
21	CH	Fat clay	23.6	2.10	1.70	71.0	4.0
22	CH	Fat clay	28.9	1.79	1.39	72.5	2.8
23	CH	Fat clay	24.6	1.59	1.28	46.0	8.8
24	CH	Fat clay	23.6	2.02	1.63	48.5	4.5
25	CH	Fat clay	19.1	1.71	1.44	95.0	1.6
26	CH	Fat clay	16.4	1.52	1.31	45.0	4.1
27	CH	Fat clay	18.8	2.21	1.86	72.0	3.1
28	CL	Lean clay	17.1	1.89	1.61	34.5	4.9
29	CH	Fat clay	24.4	1.62	1.30	49.0	4.3
30	CH	Fat clay	27.5	1.89	1.48	77.5	1.8
31	SC	Clayey sand	15.6	1.91	1.65	27.3	7.0
32	CH	Fat clay	31.2	1.89	1.44	56.5	3.4
33	CH	Fat clay	25.9	1.90	1.51	33.5	6.0
34	CH	Fat clay	26.3	1.88	1.49	51.8	4.4
35	MH	Elastic silt	28.4	1.79	1.39	55.5	3.6
36	CH	Fat clay	29.7	1.81	1.40	36.5	5.0
37	ML	Silt	29.7	1.82	1.40	110.0	3.8
38	CH	Fat clay	27.1	1.79	1.41	90.0	2.9
39	CL	Lean clay	19.8	1.91	1.59	43.0	5.5
40	CH	Fat clay	27.9	1.72	1.34	73.5	2.7
41	MH	Elastic silt	31.8	1.81	1.37	38.5	4.4
42	CH	Fat clay	29.7	1.92	1.48	32.9	7.0
43	MH	Elastic silt	44	1.71	1.19	48.0	3.7
44	MH	Elastic silt	41.1	1.69	1.20	53.5	4.4
45	MH	Elastic silt	15.4	1.89	1.64	32.5	8.0
46	MH	Elastic silt	26.1	1.72	1.36	45.5	4.9

## 2 Experimental design, materials and methods

### 2.1 Study area description

The city of Sincelejo, capital of the Department of Sucre, Subregional Center of the Colombian Caribbean urban system, is located in the northeast of the country at 9° 18″ latitude north, 75° 23″ west latitude of the Greenwich meridian; it has a total area of 28,504 hectares, of which 8% corresponds to urban territory and the remaining 92% to rural territory, with a height above mean sea level of 213 masl [1]. The soil of the municipality of Sincelejo is typical of the mountain landscape. It consists of irregular and complex relief surfaces, with variable slope and altitudes ranging from 50 to 260 m. It comprises the types of reliefs called hogbacks, bars and ridges consisting of limestone and limestone sandstone materials [2].

### 2.2 Material and methods

A total of 46 representative samples were collected from different sites in the city of Sincelejo and analyzed in the laboratory, to determine their physical and mechanical properties. In the geotechnical research sites, excavations were carried out up to a maximum depth of 1.50 m, to obtain the disturbed and undisturbed samples in the CBR cylinders for their respective testing in the laboratory and at each site chosen, field measurements were performed with the Dynamic Cone Penetrometer (DCP) test. The DCP consists of a 16 mm steel rod, to which a tempered steel cone with a 20 mm base diameter and a 60 point angle is attached. The DCP is driven into the soil by a 8 kg hammer with a dropping height of 575 mm [3]. This equipment is used for the determination of soil strength profiles under the density and moisture content conditions in their natural state. The soil samples collected were taken to the laboratory, following standard procedures to preserve their original moisture and density conditions. In the case of disturbed samples, some of these were split and dried in the oven, and then performed the soil particle size analysis tests [4] and the Atterberg limits [5] (liquid limit with Casagrande equipment and limit plastic through manual roll realization). These values allowed soil classification to be found based on AASHTO Soil Classification System [6] and Unified Soil Classification System (USCS) [7]. For the rest of the disturbed samples, natural moisture content was determined. The undisturbed soil samples collected from CBR moulds, after prior preparation, their respective wet and dry unit weights were determined, and then were failed following the standardized California Bearing Ratio (CBR) laboratory test procedure [8]. Once all the data from the field and laboratory test results was collected, the information was organized using the Excel tool.

## Declaration of Competing Interest

The authors declare that they have no known competing financial interests or personal relationships that could have appeared to influence the work reported in this paper.

## Appendix Supplementary materials

Image, application 1

Image, application 2
